# Synthesis of Biogenic Gold Nanoparticles from *Terminalia mantaly* Extracts and the Evaluation of Their In Vitro Cytotoxic Effects in Cancer Cells

**DOI:** 10.3390/molecules25194469

**Published:** 2020-09-29

**Authors:** Michele S. Majoumouo, Jyoti R. Sharma, Nicole R. S. Sibuyi, Marius B. Tincho, Fabrice F. Boyom, Mervin Meyer

**Affiliations:** 1 Antimicrobial & Biocontrol Agents Unit, Laboratory for Phytobiochemistry and Medicinal Plants Studies, Department of Biochemistry, University of Yaoundé 1, Yaoundé PO. Box 812, Cameroon; 3770612@myuwc.ac.za (M.S.M.); ffefe@yahoo.com (F.F.B.); 2 Department of Science and Innovation (DSI)/Mintek Nanotechnology Innovation Centre, Biolabels Node, Department of Biotechnology, University of the Western Cape, Private Bag X17, Bellville 7535, South Africa; Jyt228@gmail.com (J.R.S.); nsibuyi@uwc.ac.za (N.R.S.S.); 3173772@myuwc.ac.za (M.B.T.)

**Keywords:** antitumor, gold nanoparticles, green synthesis, nanotechnology, *Terminalia mantaly*

## Abstract

Scientists have demonstrated the potential of plant materials as ‘green’ reducing and stabilizing agents for the synthesis of gold nanoparticles (AuNPs) and opened new ecofriendly horizons to develop effective and less harmful treatment strategies. The current study demonstrated the use of *Terminalia mantaly* (TM) extracts to synthesize AuNPs with enhanced cytotoxic effects. The TM-AuNPs were synthesized at 25 and 70 °C using water (_W_TM) and methanolic (_M_TM) extracts of the leaf, root and stem/bark parts of the plant. The TM-AuNPs were characterized using UV–visible spectrophotometry, dynamic light scattering (DLS), transmission electron microscopy, energy dispersive X-ray (EDX), selection area electron diffraction (SAED) and Fourier transform infrared (FTIR) spectroscopy. Majority of the TM-AuNPs were spherical with a mean diameter between 22.5 and 43 nm and were also crystalline in nature. The cytotoxic effects of TM-AuNPs were investigated in cancer (Caco-2, MCF-7 and HepG2) and non-cancer (KMST-6) cell lines using the MTT assay. While the plant extracts showed some cytotoxicity towards the cancer cells, some of the TM-AuNPs were even more toxic to the cells. The IC_50_ values (concentrations of the AuNPs that inhibited 50% cell growth) as low as 0.18 µg/mL were found for TM-AuNPs synthesized using the root extract of the plant. Moreover, some of the TM-AuNPs demonstrated selective toxicity towards specific cancer cell types. The study demonstrates the potential of TM extracts to produce AuNPs and describe the optimal conditions for AuNPs using TM extracts. The toxicity of some the TM-AuNPs can possibly be explored in the future as an antitumor treatment.

## 1. Introduction

Traditional medicine has always used and relied on the medicinal properties of plants for the treatment of numerous diseases such as bacterial infections, headaches, hypertension, jaundice, leprosy and cancer, among others [1]. Among various plant species used in Cameroonian traditional medicine, *Terminalia mantaly* (TM) has gained scientific recognition due to its reported antitumor, antidiabetic and antihypertensive medicinal properties. TM is a deciduous tree belonging to the flowering plant family, Combretaceae. The genus *Terminalia* comprises of more than 100 species. Numerous scientific studies have explored the antimicrobial, antiprotozoal, antidiarrheal, anti-inflammatory, antitumor and wound healing activities; which can be attributed to phytochemicals present in the plant [1]. A survey of the literature has revealed that the *Terminalia* genus, TM included; possesses a variety of bioactive phytochemical constituents, such as tannins, pentacyclic triterpenes, glycoside derivatives, flavonoids and phenolic compounds [2]. 

Green nanotechnology has been applied to synthesize metallic nanoparticles (NPs) from medicinal plants extracts. It has been shown that the phytochemical constituents present in plants can act as reducing and capping agents during the synthesis biogenic metallic NPs, including gold nanoparticles (AuNPs) [3,4]. Several studies have demonstrated the synthesis of biogenic AuNPs using various species of the genus *Terminalia,* which include *T. catappa*, *T. chebula*, *T. arjuna* and *T. bellirica* [5,6,7,8]. While TM has been used to produce biogenic silver nanoparticles [3], this species has not been used to produce AuNPs. The conventional physical and chemical methods used for AuNP synthesis involve the use of toxic chemicals, high energy processes and are expensive [9,10,11]. Studies have highlighted concerns regarding chemically synthesized NPs for biomedical applications due to the use of toxic reducing agents such as sodium citrate, sodium borohydride and byproducts formed during the synthesis process [11,12]. These chemicals are not only corrosive but also generate flammable by products during chemical synthesis. Considering the threats these nanomaterials and the processes used to synthesize these materials pose to the environment and humans, there is a need for the development of more biofriendly nanomaterials using processes that are also more environmentally safe [11].

Scientists have turned to green nanotechnology to produce metallic NPs using biological systems (e.g., microbes and plant extracts) [11,12,13]. Since biologicals are used in the synthesis process, the expectation is that the nanomaterials will be more biofriendly and the synthesis processes would be more environmentally friendly. This eliminates the use of toxic reducing, stabilizing and capping agents and produces nanoparticles that will have shape and size-dependent biological activities [11,14,15]. Moreover, utilizing plant extracts as reducing agents for the synthesis of AuNPs is advantageous owing to the availability of the plants and simplicity of the approach. Plant-mediated synthesis of AuNPs will not only allow large-scale production but will also reduce cost and time involved in the synthesis [16].

The use of nanotechnology to produce metallic nanostructures has gained interest, especially in medicine [17,18,19]. The fascinating and insightful use of optical, chemical and catalytic properties of noble metal NPs depends on the metal source, nanoparticle sizes and shapes and surface chemistry. Furthermore, these properties allow NPs to gain impetus in the present century and make them an excellent resource for diverse applications [20,21,22,23]. Metallic NPs especially AuNPs have attracted a great deal of attention, owing to their tunable optical and electronic properties [24]. AuNPs have been reported as safe for use in drug delivery system in in vitro and in vivo studies [25]. While the antibacterial activities of some of the biogenic AuNPs produced from *Terminalia* species have been studied, not much is known about the in vitro and in vivo cytotoxicity of these AuNPs. The toxicity of AuNPs synthesized using a water extract of fruit pericarp of *T. bellirica* was tested using the brine shrimp assay and did not show any toxicity [26].

Nanotechnology can play an important role in medicine, specifically in the early disease detection, improved diagnosis, and development of personalized treatments for chronic and infectious diseases [27]. Biocompatible AuNPs have been widely studied and applied in the diagnosis and treatment of cancer [28]. In addition, several studies indicated the intrinsic antitumor property of AuNPs that were able to selectively kill cancer cells [29]. Previous reports described AuNPs that have strong antitumor effects through the induction of apoptosis in colorectal, breast and liver cancer cells [30]. Owing to their small size, AuNPs are well suited for delivering antitumor drugs due to their preferential accumulation at tumor sites through the enhanced permeability retention effect [31].

Herein we describe for the first time the synthesis of biogenic AuNPs from the leaf, root and stem/bark parts of the TM. We evaluated cytotoxic effects of biogenic AuNPs synthesized from six different TM extracts in three human cancer (Caco-2, HepG2 and MCF-7) and one non-cancer (KMST-6) cell lines and found that the AuNPs display selective toxicity towards certain cancer cell lines.

## 2. Results and Discussion

AuNPs have been applied in the development of biosensors, pharmaceuticals, nanoscience and nanotechnology, hence, the term nanomedicine was coined. In nanomedicine, AuNPs can be used as contrast and drug delivery agents for diagnostics and therapeutics, respectively. Traditionally, AuNP are synthesized through physical and chemical methods, these methods are usually limited by their use of toxic chemicals as reducing agents. To overcome the drawbacks of these methods, many studies have opted to use a green approach to synthesize AuNPs [5,32,33]. Among the green sources used, plant extracts are readily available, providing an easy and simple method that involves just one step synthesis. Moreover, utilizing plant extracts as reducing agents in synthesizing AuNPs reduces production time and cost, and offers an opportunity for large-scale production [34]. Biogenic AuNPs have been reported to elicit potent toxic effects and antiproliferative activity against various tumors [35]. The inhibitory mechanism of nanoparticles against cancer cell lines is not well known. However, it was suggested that nanoparticles can block the activity of abnormal signaling proteins or interact with functional groups of intracellular proteins and enzymes, as well as with the nitrogen bases in the DNA molecules, which results in cell death [36]. The current study explored and demonstrated the synthesis of biogenic AuNPs using _W_TM and _M_TM extracts as both reducing and stabilizing agents, and tested their in vitro cytotoxicity.

### 2.1. Qualitative Analysis of TM Phytochemicals

The phytochemical composition of TM extracts in Table 1, revealed an unequal distribution of different secondary metabolites in _W_TM and _M_TM extracts. The alkaloids, flavonoids, glucosides and total phenols were present in all the extracts, and no anthocyanins were found in any of the TM extracts. The _W_TM extracts contained majority of the phytochemicals. All the _M_TM extracts lacked steroids. Tannins and triterpenes were not present in TMSB and TMR, and TMR and TML lacked anthraquinones. Metabolites that contain active functional groups, such as hydroxyl, aldehyde and carboxyl units, may play pivotal roles in chemical reduction processes of the gold precursor to produce AuNPs [5].

### 2.2. Green Synthesis of AuNPs

**Figure 1:**
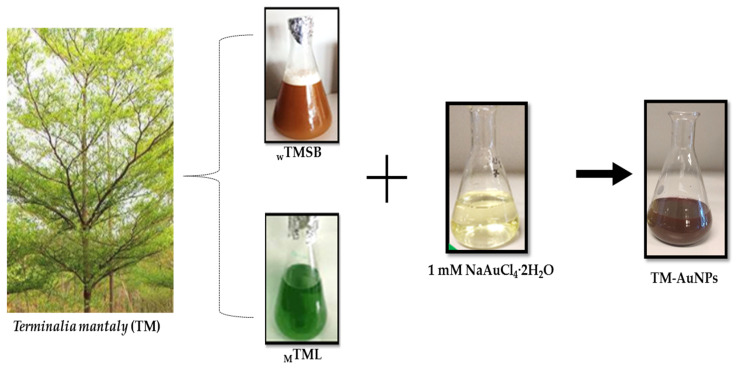
One step synthesis of TM-gold nanoparticles (AuNPs) by the reduction of chloroaurate ions by TM extracts. The _W_TM and _M_TM extracts of leaf, root and stem/bark parts of the plant were prepared and mixed with NaAuCl_4_·2H_2_O. The appearance of a red wine/ruby red color indicated the formation of the AuNPs.

Small scale AuNP synthesis was carried out in a 96 well microplate by incubating a fixed concentration (1 mM) of NaAuCl_4_·2H_2_O with increasing concentrations (0.39–12.5 mg/mL) of the 6 TM extracts (_W_TMSB, _W_TMR, _W_TML, _M_TMSB, _M_TMR and _M_TML). The formation of TM-AuNPs was indicated by a color change in the reaction mixture as is shown in Figure 1. The appearance of a red wine/ruby red color after the addition of TM extracts to the NaAuCl_4_·2H_2_O solution is an indication of AuNP synthesis. Synthesis was performed for 5 h at 25 °C and 70 °C. These temperatures are often used for the synthesis of AuNPs [18]. The red wine color, which AuNP solutions typically exhibit, is due to the excitation of surface plasmon vibrations in the AuNP solution, and indicates the formation of AuNPs [5,35,37]. TM-AuNPs were successfully synthesized with all six extracts at both 25 °C and 70 °C. In the one step reaction, the phytochemicals in the TM extracts acted as the reducing, stabilizing and capping agents. The bioreduction of the gold precursor could be ascribed to phytochemicals such as alkaloids, flavonoids, tannins, steroids and triterpenes that have been shown to be present in the plant extracts (Table 1) as was suggested in previous studies [32,33]. In particular, it is phytochemicals that contain hydroxyl, aldehyde and carboxyl functional groups that can partake in the bioreduction process [38]. Due to the scavenging capabilities of their –OH groups in phenols, these phytochemicals have been reported to be involved in the bioreduction and stabilization of NPs [33]. The phenolic compounds along with the water domains can synergistically interact with the existing nuclei and lay the foundation to create highly structured sheets of zerovalent gold [23].

#### 2.2.1. Optimization of TM-AuNP Synthesis and UV–Vis Spectroscopy

**Figure 2:**
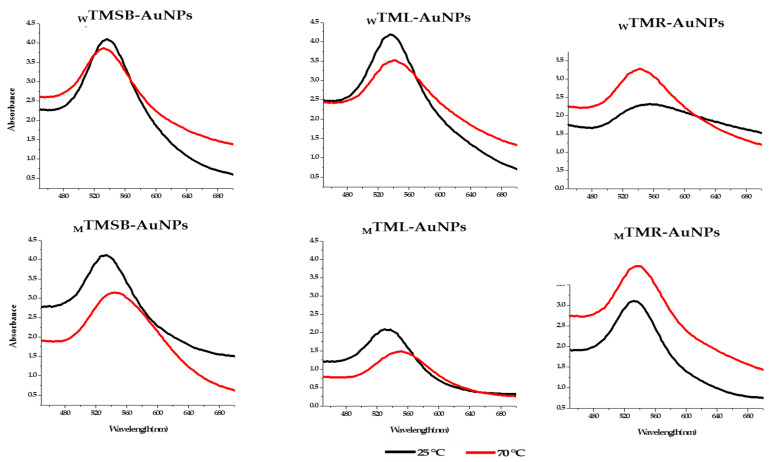
The UV–visible spectra of TM-AuNPs. The AuNPs were synthesized at the optimal concentration (OC) of TM-extracts at 25 and 70 °C using _W_TM and _M_TM extracts.

The synthesis of TM-AuNPs was further confirmed by UV–visible spectrophotometry analysis and this data was used to determine the optimum plant concentration to produce the TM-AuNPs from each of the six TM extracts. The Surface Plasmon Resonance (SPR) of AuNPs results in an absorption maxima (or λ_max_) in the region of 500–600 nm [10,39] as is illustrated in Figure 2, which shows the UV–visible spectra for TM-AuNPs produced with the six different TM extracts at 25 °C and 70 °C. These spectra also represent the AuNPs synthesized with the optimal plant extract concentrations. Table 2 indicates the optimal concentration (OC) of the plant extract and the corresponding λ_max_ for the respective TM-AuNPs (_W_TMSB-AuNPs, _W_TMR-AuNPs, _W_TML-AuNPs, _M_TMSB-AuNPs, _M_TMR-AuNPs and _M_TML-AuNPs) at both 25 °C and 70 °C. Based on the UV-visible spectrophotometry analysis, the TMR produced AuNPs with a lower peak height or optical density, which is an indication that either the nanoparticle concentration is lower or larger in size [40,41]. Figure 2 also shows that the temperature at which the synthesis was carried out significantly influenced the UV–visible spectra and characteristics of the AuNPs. The same TM extract produced a very different spectrum at the two different temperatures. For example, the optical density of TM-AuNP produced with the _W_TML extract produced at 25 °C was significantly higher than the optical density produced for the same extract at 70 °C. The shape of the spectra, which is an indication of the size, shape and uniformity of the TM-AuNPs were also different. While the λ_max_ for _W_TML-AuNP produced at 25 and 70 °C was the same (544 nm), the λ_max_ values from the other five extracts were very different at the two temperatures. This all suggests that the concentration of the extract, the phytochemicals and the temperature at which the synthesis was performed all play a role in the synthesis of the AuNPs.

#### 2.2.2. DLS Analysis of TM-AuNPs

The size distribution, charge and surface chemistry of the AuNPs are particularly important since these physicochemical properties strongly influence the mobility and bioavailability of nanoparticles when applied in biological conditions [10,11]. These characteristics can be used to predict the behavior of AuNPs in various biological environments, which is important when designing nanomaterials for biomedical applications. The TM-AuNPs synthesized at 25 and 70 °C had a hydrodynamic diameter ranging from 39 to 79 nm as shown in Table 3. Except for the _M_TML, the other five extracts produced larger AuNPs at 70 °C than at 25 °C. The size of the TM-AuNPs increased on average by 9 nm when the synthesis was done at 70 °C. It is possible that the reducing and capping agents are modified at higher temperatures and that the phytochemicals that are involved in the synthesis process at 70 °C differ from the phytochemicals present at 25 °C [42]. An independent study also reported that the sizes of AuNPs produced from plant extracts were significantly smaller at low temperatures [43].

The polydispersity index (Pdi) gives an indication of the degree of uniformity of the size distribution of a nanoparticle in solution. Pdi values above 0.7 indicate that the sample has a very broad particle size distribution [44], and that the nanoparticles may possibly also be aggregated. Except for _W_TMR-AuNPs produced at 70 °C, which had a Pdi of 0.8, the Pdi values for all the other TM-AuNPs were less or equal to 0.7 (Table 3), suggesting that these samples have an acceptable level of uniformity. TM-AuNPs with Pdi values less or equal to 0.7 were likely to be monodispersed, uniform in size and shape and stable in colloidal form.

Nanoparticles are known to agglomerate in the presence of salts due to a reduction in the electronic double layer around each particle, allowing for adhesion through van der Waals forces [45,46]. Aggregation can happen because phytochemicals present in the reaction solution are absorbed on the surface of the AuNPs, resulting in the formation of crosslinks in between the AuNPs.

Zeta potential provides pivotal information on the dispersion of nanoparticles as the magnitude of the charge and indicates the mutual repulsion between particles [47]. Nanoparticles with a zeta potential between 30 and −30 mV are more stable in solution [48] and will repel each other and they tend not to form aggregates in solution [47,49,50]. The zeta potential values for 7 of the 12 TM-AuNPs samples were within this range (Table 3). This included _W_TMSB-AuNPs synthesized at both 25 °C and 70 °C, _M_TMSB-AuNPs synthesized at 70 °C, _W_TMR-AuNPs synthesized at 70 °C, _M_TMR-AuNPs synthesized at 25 °C, and _M_TML-AuNPs synthesized at both 25 °C and 70 °C. Based on the results, these TM-AuNPs were expected to be very stable in solution, while the other five samples that had zeta potentials less than −30 mV might be prone to aggregation. These samples include _M_TMSB-AuNPs synthesized at 25 °C, _W_TMR-AuNPs synthesized at 25 °C, _M_TMR-AuNPs synthesized at 70 °C and _W_TML-AuNPs synthesized at both 25 °C and 70 °C.

The study shows that temperature greatly influenced the hydrodynamic size, charge and size distribution of the AuNPs. Using the same extract at two different temperatures produced AuNPs with very different physicochemical properties. This is further proof that the phytochemicals involved in the synthesis process vary between the different TM-AuNPs. The _W_TMR-AuNPs had a Pdi of 0.8, indicating that these nanoparticles might aggregate over time. However, an increase in the temperature at which synthesis was done for _M_TMR-AuNPs not only change size but resulted in polydispersed nanoparticles as reflected by a change in Pdi from 0.4 at 25 °C to 0.5 at 70 °C. Nanoparticles with low Pdi value are likely to be monodispersed [46], thus, TM-AuNPs that gave the lowest Pdi values may be of uniform size, shape and stable in its colloidal form.

#### 2.2.3. HRTEM, SAED and EDX Analyses

HRTEM analysis showed that the TM-AuNPs display a variety of geometrical shapes, as shown in Figure S1. Most of the TM-AuNPs were spherical in shape with some triangular, hexagonal and pentagonal shapes. Obtaining AuNPs with a variety of geometrical shapes is very common for NPs produced through plant-mediated synthesis [46]. This is speculated to be due to the presence of different phytochemicals in the extracts that might act in synergy to reduce the gold ions and form AuNPs [51]. Polyphenols have been reported to produce NPs with different shapes [5]. Biomolecules that contains highly polar groups (e.g., −OH) on their surface may increase the rate of nucleation and induce AuNP formation. The TM-AuNPs had well-defined edges and most of them were well dispersed. Based on the HRTEM analysis, the _M_TML-AuNPs, _W_TML-AuNPs and _W_TMR-AuNPs appears to be agglomerated. However, HRTEM analysis is not the best test to use to determine NP aggregation. Further analysis of the stability of the TM-AuNPs over time is needed.

**Figure 3:**
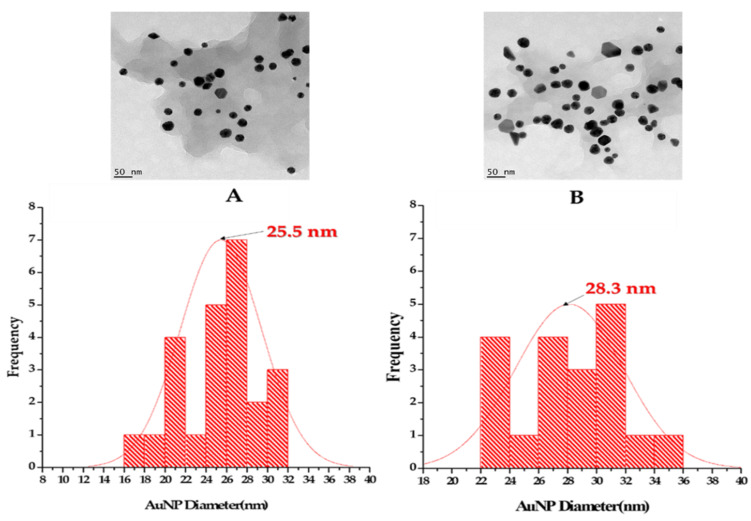
HRTEM analysis of shape and size distribution of _M_TMSB-AuNPs synthesized (**A**) at 25 °C and (**B**) 70 °C.

The size distribution of TM-AuNPs was calculated from the HRTEM micrographs, the representative histograms (Figure 3) demonstrate that _M_TMSB-AuNPs synthesized at 25 °C and 70 °C had a core size of 25.5 nm and 28.3 nm, respectively. The TM-AuNPs had a core size ranging from 21.5 to 43 nm as shown in Table 4. The _W_TML-AuNPs produced at 70 °C had a smaller core size (21.5 nm) when compared to others. This might suggest that TML might be richer in reducing and capping agents. These results were comparable to those reported by Ankamwar on AuNPs synthesized from leaf extract obtained from T. Catappa. The NPs were also spherical in morphology, with an average core size of 21.9 nm [5]. The core sizes of _W_TMSB and _W_TMR-AuNPs were bigger when compared to the sizes of the other TM-AuNPs.

To highlight the crystalline nature of nanoparticles, SAED analysis was performed. The fringe lattice values ranged from 0.167 to 0.257 nm and the SAED pattern (Figure S2, Supplementary data), which confirmed the crystalline nature of the TM-AuNPs varied between AuNPs synthesized at 25 °C and 70 °C. _M_TMSB-AuNPs synthesized at 70 °C for example had a typical HRTEM image with clear lattice fringes. Furthermore, a d-spacing or interplanar distance of 0.233 nm was obtained for TM-AuNPs, which was comparable with 0.2355 nm, corresponding to the (111) planes of face-centered cubic (fcc) gold single crystals. The clear lattice fringes in HRTEM images and the typical SAED pattern with bright circular rings corresponding to the (111), (200), (220) and (311) planes were obtained in most TM-AuNPs. This was an indication that the nanoparticles obtained were highly crystalline, confirming the fcc crystalline geometry of AuNPs (JCPDS file no. 4-0783) [52]. The diffraction patterns of TM-AuNPs were also comparable to the AuNPs synthesized from *T. catappa* leaf extracts, which showed the Bragg reflections corresponding to the (111), (200), (220), (311) and (222) sets of lattice planes [5,18]. This may be indexed based on the fcc structure of gold. However, the lattice plane was predominantly (111)-oriented. The amorphous effect (diffuse rings) was observed with _W_TMSB and _M_TMR AuNPs at 70 °C, and the Bragg reflections were weak and considerably broadened relative to the intense (111) and (200) reflections. Finally, the crystallinity was more pronounced for AuNPs synthesized at 25 °C compared to the ones produced at 70 °C.

The EDX spectra of TM-AuNPs confirmed the presence of gold ions in all the AuNPs (Figure S2, Supplementary data). The Au peaks were acquired around 2.3 keV, 9.7 keV and 11.3 keV. In some AuNPs, the EDX spectrum showed the presence of silicone (Si), which might be due to a high degree of crystallinity. These results further confirmed the SAED patterns in Figure S3 (Supplementary data). Moreover, the presence of elements such as calcium and potassium can be due to the micronutrients in the TM extracts used in the synthesis. The weak signals for oxygen in the spectra may have originated from the biomolecules bound to the surface of the NPs [52], Cu from the support HRTEM grid and film and Co from the lenses of the microscope [16].

#### 2.2.4. FTIR Analysis of TM-AuNPs

FTIR analysis was carried out to identify the possible functional chemical bonds from the phytochemicals in the TM extracts that are responsible for reduction, capping and stabilization of AuNPs [53]. The representative FTIR spectra of TMR and TMR-AuNPs are shown in Figure S4 (Supplementary data), highlighting some of the chemical bonds involved in NP synthesis. Generally, the chemical bonds identified in the TM extracts and AuNPs included C-O, C-H, –C=C–, H–C=O, –C≡C– and O-H (Table S1). Some of the peaks were absent in the AuNPs depending on the temperature (25 °C and 70 °C). For example, the FTIR spectra of _W_TMSB, _W_TMSB-AuNPs synthesized at 25 °C and at 70 °C showed prominent absorption bands at (1108 cm^−1^, 1347 cm^−1^, 1627 cm^−1^, 2106 cm^−1^, 2939 cm^−1^ and 3409 cm^−1^), (1048 cm^−1^, 1384 cm^−1^, 1639 cm^−1^, 2016 cm^−1^, 2920 cm^−1^ and 3717 cm^−1^) and (1123 cm^−1^, 1636 cm^−1^, 2106 cm^−1^, 2939 cm^−1^ and 3452 cm^−1^), respectively. The shoulder at 1048 cm^−1^, 1108 cm^−1^ and 1123 cm^−1^ was characteristic of C=O vibrations, while the stretch at 1347 cm^−1^ and 1384 cm^−1^ arose from the C-H methyl rock alkanes stretching but was absent on _W_TMSB-AuNPs at 70 °C. The recorded peaks at 1639 cm^−1^, 1627 cm^−1^ and 1638 cm^−1^ were due to the vibration of –C=C– stretch alkenes. In addition, the band at 2016 cm^−1^ could be attributed to the –C≡C– stretch alkynes. The broad stretching at 2920 cm^−1^ and 2939 cm^−1^ arose from the vibrations of H–C=O: C–H stretch aldehydes. Moreover, the presence of the shifted band at 3717 cm^−1^, 3409 cm^−1^ and 3452 cm^−1^, in the FTIR spectrum of _W_TMSB and _W_TMSB-AuNPs at 25 °C and _W_TMSB-AuNPs at 70 °C respectively, could be attributed to the OH groups in the alcohol or phenols groups. Moreover, the FTIR analysis showed the presence of OH and COOH chemical bonds in the TM extracts and AuNPs, which could be the most dominant in the synthesis of AuNPs [53]. These are the most commonly used groups (–OH, –COOH and long alkyl chains) for the functionalization of metal NPs, especially gold. This is due to the fact that they can ensure compatibility and stability within the environment of the NPs and can be used as a base for further chemical reactions once attached to the particle surface. The recorded FTIR spectra confirms that the chemical functional groups in the TM active metabolites acted as reducing and stabilizing agents in the synthesis of TM-AuNPs [23,54].

### 2.3. Effects of TM Extracts and AuNPs on Cancer Cells

The cytotoxicity of the TM extracts and AuNPs was evaluated on Caco-2 (human colon cancer cell line), MCF-7 (human breast cancer cell line), HepG2 (human liver cancer cell line) and KMST-6 (human skin fibroblasts) cells using the MTT assay. The results revealed that most of the TM extracts, and TM-AuNPs exerted significant cytotoxicity on the cancer cells in a dose-dependent manner. The IC_50_ values, summarized in Table 5, ranged from 0.18 to 93.73 µg/mL. This study found for the first time that methanol extracts of TM root and stem/bark were particularly toxic to the human breast cancer (MCF-7) cell line.

In general, the IC_50_ values of the TM-AuNPs were lower than that of the TM extracts, suggesting that the TM-AuNPs were more cytotoxic than the TM extracts (Table 5). The _M_TMR and _M_TMSB extracts were more toxic than the other four extracts. However, MCF-7 cells in particular were highly susceptible to the effects of _M_TMR and _M_TMSB extracts, with IC_50_ values 2.73 and 19.73 µg/mL, respectively. The cytotoxic profile of the TM extracts and TM-AuNPs did not display any particular pattern that could be related to the type of extract, the method of NP synthesis (i.e., synthesis at 25 or 70 °C) or the cell type. For example, while the IC_50_ values for _W_TML-AuNPs-25 °C and _W_TML-AuNPs-70 °C was only 5.71 µg/mL in the Caco-2 cell line, the cytotoxicity of these NPs was very different in the MCF-7 and HepG2 cell lines. The IC_50_ value for _W_TML-AuNPs-25 °C in the MCF-7 cell line was 6.56 µg/mL, while the IC_50_ value for _W_TML-AuNPs-70 °C at 32.59 µg/mL was much higher. Similarly, _M_TMR-AuNPs-25 °C and _M_TMR-AuNPs-70 °C displayed very different cytotoxicity in HepG2 cells. With an IC_50_ value of 0.18 µg/mL, _M_TMR-AuNPs-25 °C was highly toxic to HepG2 cells, but _M_TMR-AuNPs-70 °C was less toxic to these cells with an IC_50_ value of 90.85 µg/mL. The cytotoxicity of the TM-AuNPs was thus highly selective. This selectivity may be as a result of the cell type and the characteristics of the AuNPs. These characteristics were greatly influenced by the type of extract and the method of synthesis. This selective cytotoxicity is a highly desirable characteristic that can be explored for the selective destruction of specific cancer cells. Interestingly, the IC_50_ values of some of the AuNPs were significantly lower than the dose used for the positive controls. Dimethyl sulfoxide (DMSO) at 10% reduced the cell viability to 17–55%, while cisplatin reduced viability to 2–39%. DMSO was selected as a positive control as it is well known to induce cell death at high doses [55]. The proven toxicity of the solvent has led to its use as a positive control [56], while cisplatin is a clinically used chemotherapeutic antitumor drug [57].

The antitumor activity of *Terminalia* species is well known [58]. *T. ferdinandiana* fruit and leaf extracts exhibited cytotoxicity against human carcinoma cell lines including Caco-2 cells (IC_50_ = 102 µg/mL). Nandagopal et al. [58] demonstrated the cytotoxicity of *T. cheduba* seed extracts against HepG2 cells (IC_50_ = 40 µg/mL). Likewise, acetone extracts of *T. belerica* and *T. chebula* exhibited differential cytotoxic activities in several cancer cell lines, with the breast cancer cell line, MCF-7 being highly susceptible to the effects of this extract [59]. Saleem et al. [60] reported that methanol extracts of the *T. chebula* fruit reduced cell viability and inhibited cell proliferation in several malignant cell lines including MCF-7 cells. Phytochemical analysis of TM has revealed the presence of alkaloids, flavonoids, glucosides and total phenols (Table 1), these secondary metabolites were reported to have antitumor activities.

It has been demonstrated before that AuNPs exert in vitro cytotoxicity on several human cancer cells including HepG2 and triple negative breast cancer cells (MDA-MB-231) [61,62,63]. Green-synthesized AuNPs from *Cassia tora* leaf extracts demonstrated the cytotoxic efficacy against colon cancer (Col320) cell lines and not in the normal (Vero) cell lines [64]. AuNPs from *Gymnema sylvestre* leaf extracts were also investigated for their antitumor effects against HT-29 cells. The study revealed that these AuNPs exerted significant cytotoxic effects against HT-29 cancer cells at a maximal concentration of 95 μg/mL [65].

It has been reported that spherical and rod-shaped AuNPs are more efficient in reducing cell proliferation of cancer cells than AuNPs with other shapes [66]. TM-AuNPs studied here were mostly spherical, which possibly contributes to the high cytotoxicity of these AuNPs. The capping agents (phytochemicals) that play a role in the synthesis of biogenic AuNPs can also affect the cytotoxicity of AuNPs [3,5,20]. Phytochemical analysis showed that chemical composition of the various extracts was very different. This may account for the differences in the physical characteristics of the TM-AuNP as well as the differences in cytotoxicity.

The AuNPs that demonstrated higher cytotoxicity (_M_TMR-AuNPs at 25 °C and _M_TMSB-AuNPs at 70 °C) with IC_50_ ≤ 40 µg/mL on at least two cancer cell lines were selected for evaluating the cytotoxicity with non-cancerous fibroblast KMST-6 cells. The CC_50_ values (cytotoxic concentration of the AuNPs that inhibited 50% cell viability in normal cells) and the selectivity index of the selected TM-AuNPs are summarized in Table 6. The CC_50_ values of _M_TMR-AuNPs at 25 °C and _M_TMSB-AuNPs at 70 °C were 275.7 and 334.5 µg/mL, respectively. The selectivity of _M_TMR-AuNPs (25 °C) and _M_TMSB-AuNPs (70 °C) on the three cancer (MCF, HepG2 and Caco-2) cells was within the selectivity index range from 6.5 to 82.2. The results corroborate with the finding of [53] that showed that biogenic AuNPs synthesized from the South African *Galenia africana* and *Hypoxis hemerocallidea* plants extracts showed that there was no significant reduction in viability of KMST-6 cell after 24 h treatment with AuNPs with concentrations up to 32 nM [16]. The same finding has been demonstrated by Patra [67], showing that AuNPs were non-toxic towards Hek293T cells. These findings suggested that AuNPs-assisted thermotherapy could cause targeted cancer cell ablation while avoiding damage to surrounding noncancerous cells [11,67] and used in humans for drug delivery and bioimaging applications.

## 3. Materials and Methods

### 3.1. Collection and Processing of Plant Material

Mature TM leaves (TML), root (TMR) and the stem/bark (TMSB) were collected from Yaoundé (Cameroon, East Africa). The plant species was identified at the National Herbarium of Cameroon in Yaoundé, Reference number 64212/HNC. The TML, TMR and TMSB samples were washed using sterile distilled water and air dried at room temperature (25 ± 2 °C) for 3 weeks. The dried plant materials were ground into fine powders using a high-speed electrical blender and stored in a desiccator at room temperature until further analysis.

### 3.2. Plant Extraction and Phytochemical Analysis

#### 3.2.1. Preparation of Crude Extracts

The plant material was extracted using water (denoted as _W_) and methanol (denoted as _M_). Six different extracts, denoted as _W_TMSB, _W_TMR, _W_TML, _M_TMSB, _M_TMR and _M_TML, were prepared. The samples were prepared according to the following protocol: 100 g of pant material was added into 500 mL of either methanol or distilled water and incubated at room temperature (25 °C) for 48–72 h. The samples were filtered using Whatman N°1 filter paper, the residues were re-extracted under the same conditions and added to the first filtrates. The methanolic extracts were evaporated using a rotary evaporator (Büchi 011, Flawil, Switzerland) at 40 °C, the water extracts were lyophilized using a Martin Christ Beta 2-8 lyopholizer (Osterode am Harz, Germany). The dried residues were kept at 4 °C until further experiments.

#### 3.2.2. Qualitative Phytochemical Analysis

The presence of the following classes of compounds, i.e., alkaloids, flavonoids, glycosides, saponins, tannins and terpenoids, in the TM extracts was performed according to previously described standard procedures [68,69]. Briefly, 50 mg/mL of _W_TM and _M_TM were subjected to various chemicals to determine the presence of various phytochemicals. The assays or chemicals used include Mayer’s reagent (alkaloids), Shinoda test (flavonoids), Ferhling solution (glycosides), froth test (saponins), tannins (ferric chloride test), phenolic content (ferric chloride test), anthraquinones (Borntrager’s reaction test), sterols and terpenoids (Liebermann–Burchard test). The assays are qualitative and based on color change, frothing or precipitation between the active groups in the extracts and specific chemical reagents.

### 3.3. Biosynthesis of AuNPs and Characterization

#### 3.3.1. Green Synthesis of AuNPs

AuNPs were synthesized following a protocol described by Elbagory et al. [70] with slight modifications. In a 96 well polystyrene microplates, 250 µL of 1 mM of sodium tetrachloroaurate(III) dihydrate (Sigma-Aldrich, St Louis, USA) was added to 50 µL of plant extract stock solutions at varying concentrations (0.78–50 mg/mL) to a final volume of 300 µL. The solutions were incubated at 25 °C and 70 °C with orbital shaking at 40 rpm for 5 h. AuNP formation was assessed by measuring the SPR within the UV–Vis range (450–700 nm) using a POLARstar Omega microtitre plate reader (BMG Labtech, Germany). AuNP synthesis was scaled up to 2 mL following the optimum conditions.

#### 3.3.2. Characterization of TM-AuNPs

The TM-AuNPs were characterized by UV–visible spectroscopy, dynamic light scattering (DLS), high-resolution transmission electron microscopy (HRTEM), energy-dispersive X-ray spectroscopy (EDX) and the selected area electron diffraction (SAED) analyses.

##### UV–Visible Spectroscopy

Formation of TM-AuNPs was preliminarily confirmed by visual observation for color change to red wine, further by UV–visible spectra after 5 h synthesis. Sharp peak obtained from the UV–visible spectrum confirmed the presence of AuNP at the absorption range between 450 and 700 nm using a POLAR star Omega microplate reader (BMG labtech, Offenburg, Germany).

##### DLS Analysis

The TM-AuNPs were washed 3 times with distilled H_2_O and centrifuged at 10,000 rpm for 10 min, the NPs were resuspended in double distilled H_2_O. The AuNPs were analyzed by DLS to measure their hydrodynamic size, zeta potential and Pdi using a Zetasizer (Malvern Instruments Ltd., Malvern, UK).

##### TM-AuNPs HRTEM, EDX and SAED Pattern Analysis

The structure, size distribution, composition, presence of reducing Au^3+^ ions and crystallinity form of TM-AuNPs were analyzed by the HRTEM using a FEI Tecnai G2 20 field-emission HRTEM (Oregon, OR, USA). Additionally, the HRTEM was also used for EDX and SAED analyses. The samples were prepared by drop-coating one drop of each sample onto a carbon-coated copper grid. The AuNPs were dried under a Xenon lamp for 10 min and analyzed by HRTEM. Transmission electron micrographs were captured in the bright field mode at an accelerating voltage of 200 KeV. EDX spectra were collected using an EDX liquid nitrogen cooled lithium doped silicon detector. The TEM micrographs were analyzed using origin 8.5 and Image J Software (50b version 1.8.0_60, http://imagej.175nih.gov/ij).

##### FTIR Spectroscopy Measurements

The FTIR spectra were obtained from JASCO 460 plus spectrophotometer (Perkin Elmer, Massachusetts, MA, USA) with KBr at a frequency ranging from 4000 to 400 cm^−1^ in a KBr matrix. The TM-AuNPs were centrifuged at 10,000 rpm for 10 min and dried at 70 °C using an oven for 2 min. The TM extracts and AuNPs powders were individually mixed with KBr powder and pressed into a pellet for measurement. Background correction was made using a reference blank KBr pellet. The baseline corrections were performed for all spectra.

### 3.4. Cell Viability Using MTT Assay

The viability of the MCF 7, Caco-2, HePG2 and KMST-6 cells treated with TM crude extracts and AuNPs was evaluated using the MTT assay as described previously with some modifications [71]. The cells were maintained in DMEM containing 10% fetal bovine serum and 1% penicillin–streptomycin cocktail in a 37 °C humidified incubator with 5% CO_2_ saturation. The cells were seeded in 96-well microtitre plates at a density of 5 × 10^5^ cells/100 μL/well. After 24 h, the culture medium was replaced with fresh medium containing the TM extracts and AuNPs at increasing concentrations. The stock solutions of TM-AuNPs were prepared in ddH_2_O at 1 mg/mL, and for the TM-extracts (10 mg/mL) were prepared in 10% DMSO. Two-fold dilutions of the samples from 7.8125–1000 µg/mL were added to the 96-well plate containing various cell lines. Untreated cells were used as a negative control, and cells were treated with 10% DMSO and 100 µg/mL of cisplatin (Sigma-Aldrich) as positive controls. For the AuNP interference test, cells treated with increasing concentrations of TM-AuNPs were left without the MTT dye. All treatments were done in triplicate. After 24 h, the media were removed in all wells. Thereafter, 100 μL of the MTT reagent (prepared from 5.0 mg/mL stock solution) was added to each well. The cells were incubated at 37 °C for 4 h then the MTT reagent was replaced with 100 μL of DMSO to dissolve the purple formazan crystals and incubated for a further 30 min. The absorbance of the formazan product formed was measured at a 570 nm wavelength (λ), with background subtracted at the λ of 700 nm using a PolarSTAR Omega plate reader. The percentage of cell viability was calculated by comparing the absorbance of the test samples with the absorbance of the control (untreated) samples, multiplied by 100%. The data represent the average means of triplicate measurement from three independent experiments. The IC_50_ for cancer cells and CC_50_ for non-cancer cells values were determined using GraphPad Prism version 8.0.0, GraphPad Software, San Diego, California USA. The degree of selective toxicity of the active TM-AuNPs towards cancer cell lines relative to the non-cancer cell line (KMST-6) was expressed as the selectivity index (SI) and calculated as follows:

$$\mathrm{Selectivity}\;\mathrm{Index}\;\left(\mathrm{SI}\right)=\frac{\mathrm{CC}50\;\mathrm{in}\;\mathrm{non}-\mathrm{cancer}\;\mathrm{cells}\;\left(\mathrm{KMST}-6\;\mathrm{cells}\right)}{\mathrm{IC}50\;\mathrm{in}\;\mathrm{cancer}\;\mathrm{cells}}$$

### 3.5. Statistical Analysis

The data were from at least three independent experiments and analyzed using a one-way ANOVA and Turkey post-hoc test using Graph Pad Prism. Data are expressed as mean ± SD of experiments performed in triplicate.

## 4. Conclusions

The reported synthesis is not only cost-effective but also ecofriendly and has the ability to produce stable and monodispersed AuNPs. The metabolites in the TM extracts played a key biochemical role in the synthesis, size and morphology of the AuNPs. To the best of our knowledge, this is the first report describing the potential of biomolecules from TM extracts to produce highly stable AuNPs with a small diameter range from 22.5 to 43 nm. The study also demonstrated the enhanced and selective cytotoxic properties of TM-AuNPs. The study clearly shows the potential of TM-AuNPs as antitumor agents. Further studies will be performed to isolate and characterize the phytochemicals responsible for AuNP synthesis in order to produce uniform shapes, to evaluate the possible cell death mechanism and to further explore the selective cytotoxic effects in other cancer cells.

## Figures and Tables

**Table 1:** Analysis of phytochemical composition in *Terminalia mantaly* (TM) extracts.

TM Extracts	Alkaloids	Phenolic content	Flavonoids	Tannins	Anthocyanins	Anthraquinones	Steroids	Triterpenes	Glucosides	Saponins
_W_TMSB	+	+	+	+	-	+	+	+	+	+
_M_TMSB	+	+	+	-	-	+	-	-	+	+
_W_TMR	+	+	+	+	-	+	-	-	+	+
_M_TMR	+	+	+	-	-	-	-	-	+	+
_W_TML	+	+	+	+	-	+	+	+	+	+
_M_TML	+	+	+	+	-	-	-	+	+	-

(+) Presence; (-) Absence.

**Table 2:** The OC of TM extracts for AuNP synthesis and the SPR of TM-AuNPs.

AuNPs	AuNPs at 25 °C		AuNPs at 70 °C	
	OC	λ_max_	OC	λmax
	(mg/mL)	(nm)	(mg/mL)	(nm)
_W_TMSB-AuNPs	1.56	545	1.56	540
_M_TMSB-AuNPs	1.56	550	1.56	535
_W_TMR-AuNPs	6.25	560	6.25	550
_M_TMR-AuNPs	1.56	540	1.56	550
_W_TML-AuNPs	1.56	540	1.56	545
_M_TML-AuNPs	3.12	544	1.56	544

**Table 3:** Dynamic light scattering (DLS) analysis of TM-AuNPs synthesized at 25 and 70 °C.

TM-AuNPs	AuNPs at 25 °C			AuNPs at 70 °C		
	PD (nm)	Pdi	ZP (mv)	PD (nm)	Pdi	ZP (mv)
_W_TMSB-AuNPs	39	0.5	−27	57	0.3	−28
_M_TMSB-AuNPs	44	0.5	−36	52	0.5	−29
_W_TMR-AuNPs	66	0.7	−32	79	0.8	−29
_M_TMR-AuNPs	44	0.4	−30	48	0.5	−32
_W_TML-AuNPs	43	0.6	−37	44	0.7	−35
_M_TML-AuNPs	55	0.4	−29	51	0.5	−10

Note: PD—particle diameter, Pdi—polydispersity index and ZP—zeta potential.

**Table 4:** Core sizes of TM-AuNPs were analyzed by HRTEM.

AuNPs	AuNP Core Size (nm)	
	25 °C	70 °C
_W_TMSB-AuNPs	35.5	43.0
_M_TMSB-AuNPs	25.5	28.3
_W_TML-AuNPs	26.5	21.5
_M_TML-AuNPs	23.5	25.0
_W_TMR-AuNPs	32.0	33.5
_M_TMR-AuNPs	26.0	29.5

**Table 5:** IC_50_ values of TM extracts and AuNPs against Caco-2, MCF-7 and HepG2 cells.

Treatments	IC_50_ Values (µg/mL)		
	HepG2	Caco-2	MCF-7
_W_TML	61.19 ± 0.00	90.19 ± 0.12	66.84 ± 0.01
_M_TML	75.07 ± 0.01	87.34 ± 0.00	72.44 ± 0.00
_W_TMSB	93.73 ± 0.00	62.66 ± 0.01	49.23 ± 0.00
_M_TMSB	41.28 ± 0.02	76.37 ± 0.01	19.73 ± 0.02
_W_TMR	90.47 ± 0.01	73.03 ± 0.00	43.30 ± 0.00
_M_TMR	43.24 ± 0.13	89.02 ± 0.00	2.73 ± 0.01
_W_TML-AuNPs (25 °C)	63.09 ± 0.00	5.71 ± 0.03	6.56 ± 0.01
_W_TML-AuNPs (70 °C)	41.74 ± 0.06	5.71 ± 0.31	32.59 ± 0.10
_M_TML-AuNPs (25 °C)	38.75 ± 0.01	41.20 ± 0.03	15.37 ± 0.06
_M_TML-AuNPs (70 °C)	85.07 ± 0.05	18.37 ± 0.41	54.56 ± 0.02
_W_TMSB-AuNPs (25 °C)	66.29 ± 0.01	20.34 ± 0.05	65.15 ± 0.02
_W_TMSB-AuNPs (70 °C)	63.09 ± 0.21	7.04 ± 0.01	1.77 ± 0.01
_M_TMSB-AuNPs (25 °C)	30.56 ± 0.10	75.83 ± 0.01	54.46 ± 0.01
_M_TMSB-AuNPs (70 °C)	36.26 ± 0.07	6.56 ± 0.05	1.24 ± 0.00
_M_TMR-AuNPs (25 °C)	0.18 ± 0.01	23.58 ± 0.02	3.36 ± 0.20
_M_TMR-AuNPs (70 °C)	90.85 ± 0.08	31.51 ± 0.03	6.23 ± 0.01
_W_TMR –AuNPs (25 °C)	88.59 ± 0.11	4.74 ± 0.01	1.24 ± 0.01
_W_TMR-AuNPs (70 °C)	58.03 ± 0.41	40.42 ± 0.04	6.29 ± 0.01
**% Cell Viability**			
10% DMSO	17.04 ± 0.06	34.24 ± 0.22	55.23 ± 0.14
100 µg/mL Cisplatin	20.6 ± 0.34	39.2 ± 1.67	1.9 ± 0.04

Note: The IC_50_ values were determined using GraphPad Prism. The temperatures in brackets indicate the temperature at which synthesis was carried out. Samples were compared using a one-way ANOVA and Turkey post-hoc test.

**Table 6:** Cytotoxicity of selected TM-AuNPs on KMST-6 cells and their selectivity index.

AuNPs	CC_50_ (µg/mL)	Selectivity Index (CC_50_/IC_50_)		
	KMST-6	MCF-7	HepG2	Caco-2
_M_TMR-AuNPs (25 °C)	275.7 ± 0.010	82.05 ± 0.010	1531.66 ± 0.100	11.69 ± 0.500
_M_TMSB-AuNPs (70 °C)	334.5 ± 0.020	269.75 ± 0.100	9.23 ± 0.110	50.99 ± 0.100

Note: The CC_50_ values were determined using GraphPad Prism. Samples were compared using a one-way ANOVA and Turkey post-hoc test.

## Supplementary Materials

The following are available online, Figure S1: HRTEM images of TM-AuNPs synthetized at 25 °C (A) and 70 °C (B). The arrows points at different NP shapes. Scale bar at 10and 20 nm, Figure S2: EDX spectra of TM-AuNPs synthetized at 25 and 75 °C. The green arrows show Au ions peaks, Figure S3: SAED patterns of TM-AuNPs showing single facets of NPs in TEM micrographs. The HRTEM images shows a fringe spacing of TM-AuNPs synthesized at 25 °C and 70 °C, Figure S4: FTIR spectra of TMR extracts and AuNPs synthesized at 25 °C and 70 °C, Table S1: FTIR analysis of chemicals groups in the TM extracts and AuNPs synthesized at 25 °C and 70 °C
