# Supplementary material for: Synthesis of Biogenic Gold Nanoparticles from Terminalia mantaly Extracts and the Evaluation of Their In Vitro Cytotoxic Effects in Cancer Cells
Source: Molecules. 2020 Sep 29;25(19):4469. doi: 10.3390/molecules25194469 (PMC7582329; doi:10.3390/molecules25194469)
Supplement: Supplementary file 1 [file molecules-25-04469-s001.zip › molecules-893022-supplementary.docx]

Supplementary materials

Synthesis of biogenic gold nanoparticles from *Terminalia mantaly* extracts and the evaluation of their cytotoxic effect in cancer cells.

Michele S. Majoumouo^1,2^, Jyoti R. Sharma^2^, Nicole R. S. Sibuyi^2^, Marius B. Tincho^2^, Fabrice F. Boyom ^1^, Mervin Meyer^2*†^.

^1^ Antimicrobial & Biocontrol Agents Unit, Laboratory for Phytobiochemistry and Medicinal Plants Studies, Department of Biochemistry, University of Yaoundé 1,Yaoundé, Cameroon; FFB- [ffefe@yahoo.com](mailto:ffefe@yahoo.com)

^2^ Department of Science and Innovation (DSI)/Mintek Nanotechnology Innovation Centre, Biolabels Node, Department of Biotechnology, University of the Western Cape, Bellville, South Africa, MSM- [3770612@myuwc.ac.za](mailto:3770612@myuwc.ac.za); JRS-[Jyt228@gmail.com](mailto:Jyt228@gmail.com), NRSS- [nsibuyi@uwc.ac.za](mailto:nsibuyi@uwc.ac.za), MBT-[3173772@myuwc.ac.za](mailto:3173772@myuwc.ac.za), MM- [memeyer@uwc.ac.za](mailto:memeyer@uwc.ac.za)

***** Correspondence: [memeyer@uwc.ac.za](mailto:memeyer@uwc.ac.za); Tel.: +27 21 9592032


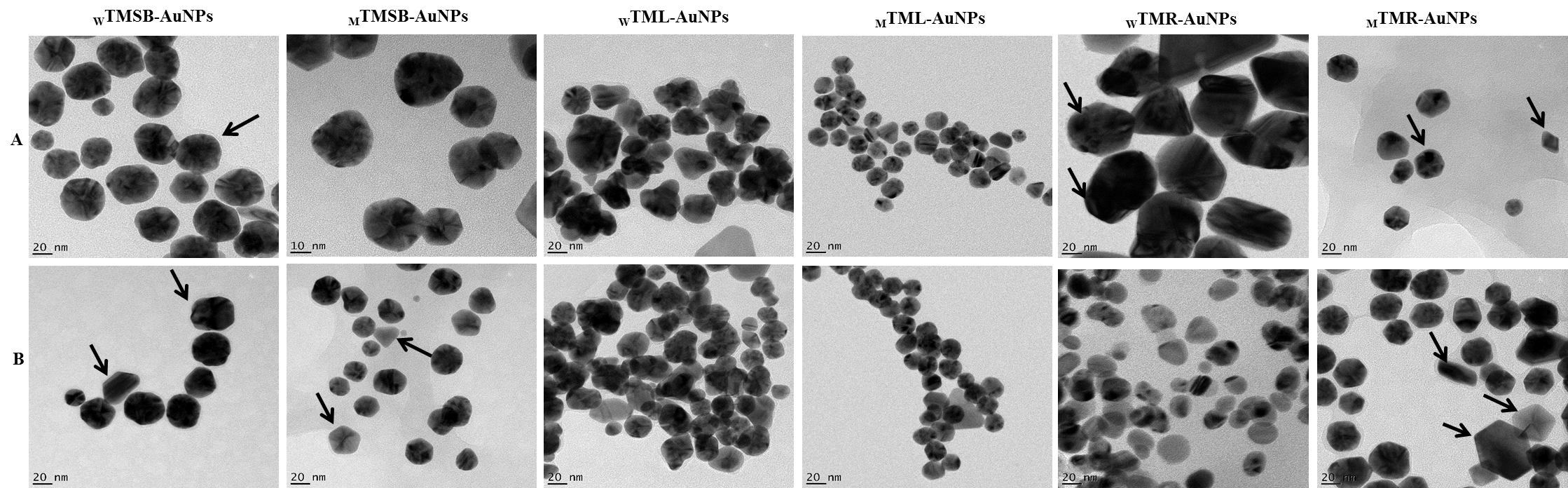


**Figure S1.** HRTEM images of TM-AuNPs synthetized at 25 °C (**A**) and 70 °C (**B**). The arrows points at different NP shapes. Scale bar at 10 and 20 nm.


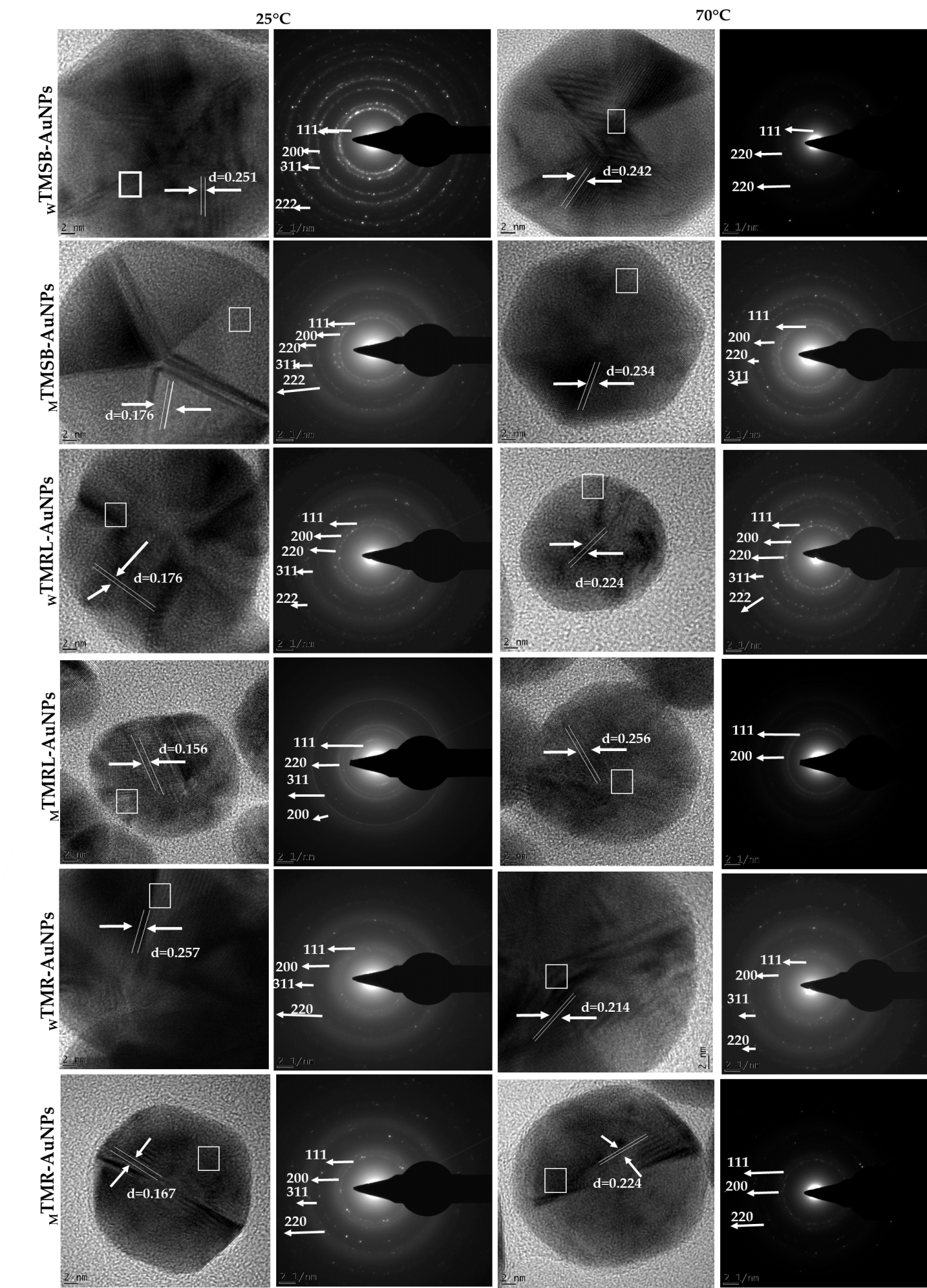


**Figure S2.** SAED patterns of TM-AuNPs showing single facets of NPs in TEM micrographs. The HRTEM images shows a fringe spacing of TM-AuNPs synthesized at 25 °C and 70 °.


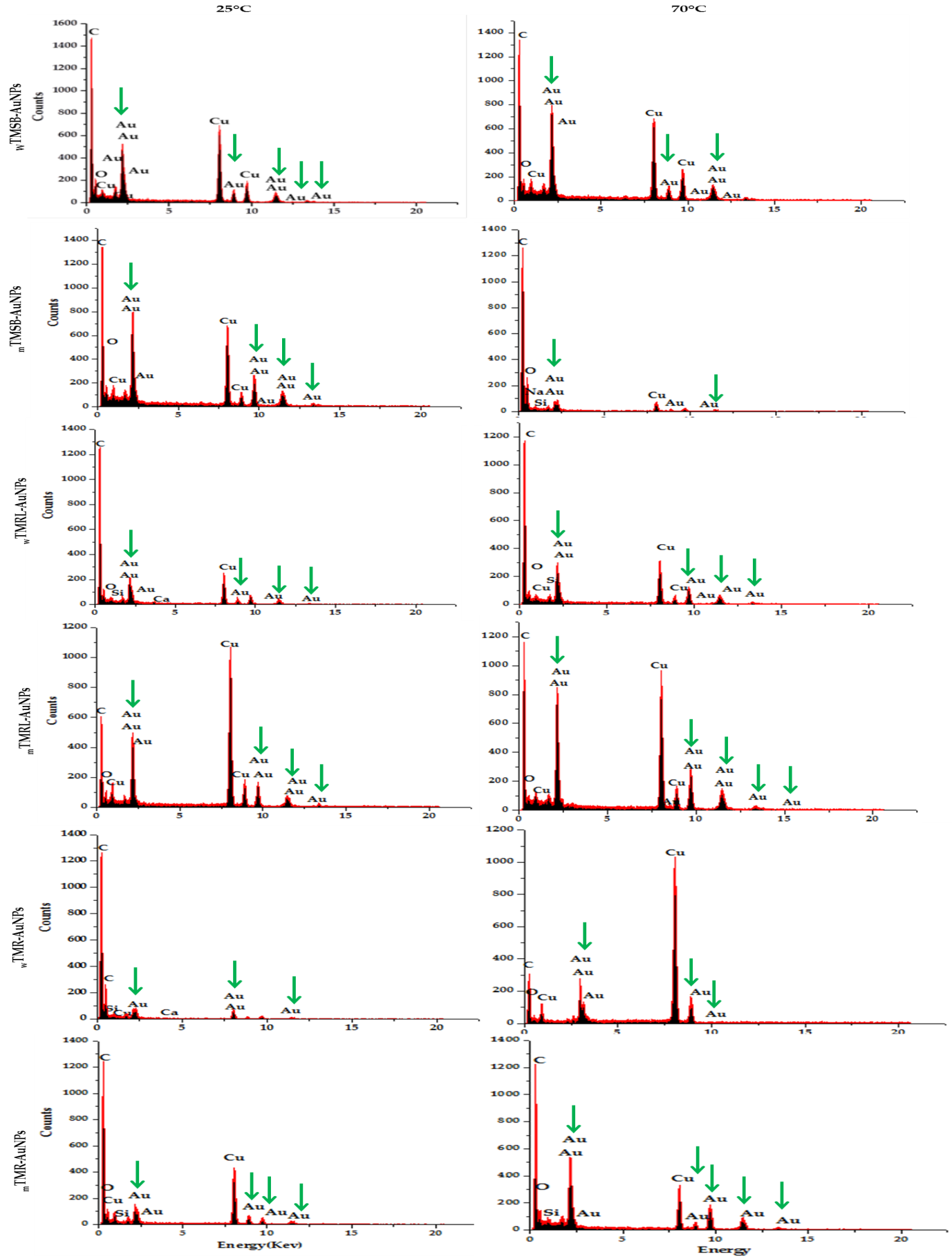


**Figure S3.** EDX spectra of TM-AuNPs synthetized at 25 and 75 ^o^C. The green arrows show Au ions peaks.

|  | **_W_TMR** | **_M_TMR** |
| --- | --- | --- |
| **AuNPs-70 ^o^C** |  |  |
| **AuNPs-25 ^°^C** |  |  |
| **Extract** |  |  |

**Figure S4.** FTIR spectra of TMR extracts and AuNPs synthesized at 25 °C and 70 °C.

**TableS1.** FTIR analysis of chemicals groups in the TM extracts and AuNPs synthesized at 25 °C and 70 °C.

| **Extracts** | **Peak position**  **in extracts**  **(cm^-1^)** | **Peak position**  **in AuNPs**  **at 25 °C(cm^-1^)** | **Peak position**  **in AuNPs**  **at 70 °C (cm^-1^)** | **Possible chemicals groups** |
| --- | --- | --- | --- | --- |
| **_W_TMSB** | 1048  1384  1639  2920  2016  3717 | 1108  1347  1627  2939  2106  3409 | 1123  -----  1636  2939  2106  3452 | C-O carboxylic acids, esters, ethers, C-H methyl rock alkanes,  –C=C– stretch alkenes,  H–C=O: C–H stretch aldehydes,  –C≡C– stretch alkynes,  O-H, Alcohol, phenol |
| **_M_TMSB** | 1044  1330  1692  2958  3645 | 1104  -----  1626  2938  3417 | 1032  1396  1638  2927  3474 | C-O carboxylic acids, esters, ethers, C-H methyl rock alkanes,  –C=C– stretch alkenes,  H–C=O: C–H stretch aldehydes,  O-H Alcohol, phenol |
| **_W_TMR** | 1044  1318  1597  3114 | 1106  1338  1626  3450 | 1101  1379  1635  3473 | C-O carboxylic acids, esters, ethers, C-H methyl rock alkanes,  –C=C– stretch alkenes,  O-H Alcohol, phenol |
| **_M_TMR** | 1044  1363  1597  2075  3156 | 1109  1334  1626  2088  3450 | 1099  1377  1637  2095  3473 | C-O carboxylic acids, esters, ethers, C-H methyl rock alkanes,  –C=C– stretch alkenes,  H–C=O: C–H stretch aldehydes ,  O-H Alcohol, phenol |
| **_W_TML** | 1129  1640  2946  3647 | 1184  1626  2828  3450 | 1132  1650  ------  3466 | C-O Aromatic esters, ethers,  –C=C– stretch alkenes,  H–C=O: C–H stretch aldehydes,  OH, Alcohol, phenol |
| **_M_TML** | 1066  1394  1635  2991  3306 | 1163  -------  1641  2991  3250 | 1066  1394  1635  2989  3304 | -O Aromatic esters, ethers,  C-H methyl rock alkanes,  –C=C– stretch alkenes,  H–C=O: C–H stretch aldehydes,  OH, Alcohol, phenol |
